# Competing risk analysis of cardiovascular disease risk in breast cancer patients receiving a radiation boost

**DOI:** 10.1186/s40959-024-00206-4

**Published:** 2024-02-09

**Authors:** Yvonne Koop, Femke Atsma, Marilot C.T. Batenburg, Hanneke Meijer, Femke van der Leij, Roxanne Gal, Sanne G.M. van Velzen, Ivana Išgum, Hester Vermeulen, Angela H.E.M. Maas, Saloua El Messaoudi, Helena M. Verkooijen

**Affiliations:** 1 Department of Cardiology, Radboud University Medical Center, Nijmegen, The Netherlands; 2 Scientific Institute for Quality of Healthcare, Radboud University Medical Center, Nijmegen, The Netherlands; 3 Division of Imaging and Oncology, University Medical Center Utrecht, Utrecht University, Utrecht, The Netherlands; 4 Department of Radiation Oncology, Radboud University Medical Center, Nijmegen, The Netherlands; 5 Department of Radiation Oncology, University Medical Center Utrecht, Utrecht University, Utrecht, The Netherlands; 6 Department of Biomedical Engineering and Physics, Amsterdam University Medical Center, Location University of Amsterdam, Amsterdam, The Netherlands; 7 Amsterdam Cardiovascular Sciences, Heart failure & arrhythmias, Amsterdam, The Netherlands; 8 Department of Radiology and Nuclear Medicine, Amsterdam University Medical Center, Location University of Amsterdam, Amsterdam, The Netherlands; 9 Informatics Institute, Faculty of Science, University of Amsterdam, Amsterdam, The Netherlands; 10 Faculty of Health and Social Studies, Research Department of Emergency and Critical Care, HAN University of Applied Sciences, Nijmegen, The Netherlands; 11 Julius Centre for Health Sciences and Primary Care, Cardiovascular Epidemiology, Utrecht Medical Centre, Utrecht University, Att. Yvonne Koop, str 6.131, P.O. Box 85500, Utrecht, 3508 GA The Netherlands; 12 Dutch Heart Foundation, The Hague, The Netherlands

**Keywords:** Radiotherapy, Breast cancer, Radiation boost, Ischemic heart disease, Cardiovascular disease, Cancer therapy-related cardiac damage

## Abstract

**Background:**

Thoracic radiotherapy may damage the myocardium and arteries, increasing cardiovascular disease (CVD) risk. Women with a high local breast cancer (BC) recurrence risk may receive an additional radiation boost to the tumor bed.

**Objective:**

We aimed to evaluate the CVD risk and specifically ischemic heart disease (IHD) in BC patients treated with a radiation boost, and investigated whether this was modified by age.

**Methods:**

We identified 5260 BC patients receiving radiotherapy between 2005 and 2016 without a history of CVD. Boost data were derived from hospital records and the national cancer registry. Follow-up data on CVD events were obtained from Statistics Netherlands until December 31, 2018. The relation between CVD and boost was evaluated with competing risk survival analysis.

**Results:**

1917 (36.4%) received a boost. Mean follow-up was 80.3 months (SD37.1) and the mean age 57.8 years (SD10.7). Interaction between boost and age was observed for IHD: a boost was significantly associated with IHD incidence in patients younger than 40 years but not in patients over 40 years. The subdistribution hazard ratio (sHR) was calculated for ages from 25 to 75 years, showing a sHR range from 5.1 (95%CI 1.2–22.6) for 25-year old patients to sHR 0.5 (95%CI 0.2–1.02) for 75-year old patients.

**Conclusion:**

In patients younger than 40, a radiation boost is significantly associated with an increased risk of CVD. In absolute terms, the increased risk was low. In older patients, there was no association between boost and CVD risk, which is likely a reflection of appropriate patient selection.

**Supplementary Information:**

The online version contains supplementary material available at 10.1186/s40959-024-00206-4.

## Background

Radiotherapy reduces the risk of recurrence after breast cancer (BC) [1, 2]. At the same time, radiation dose to the heart and vessels is associated with higher risk of ischemic heart disease (IHD) > 5 years after treatment [3–5]. It has been shown that the incidence of IHD increases linearly with 7.4% per Gray (Gy) of mean heart dose (MHD), though modern radiation regimen with cardiac sparing techniques have reduced MHD drastically [4, 6]. The specific dose to a cardiac structure, such as the dose on the left ventricle or coronary arteries, may be a more accurate predictor of IHD risk [7–9]. Furthermore, for radiation fields that are close to the heart, such as left-sided inner-quadrant tumors, the risk for cardiovascular mortality is more than twofold compared to patients with outer-quadrant tumors [10]. 

For early-stage BC patients, a radiation boost reduces local recurrence risk but does not seem to improve overall survival [11]. The benefit of a radiation boost reduces with increasing age and patients < 40 years have the largest risk reduction for local recurrences [11]. Age - combined with tumor characteristics - is an important indicator for receiving a radiation boost [12, 13]. Although, the positive effect of a radiation boost may be outweighed by an increased cardiovascular risk in certain groups, in particular in patients with an unfavorable dose distribution to cardiac structures. Evaluating the risk of cardiac toxicity for boost radiation could provide knowledge to patients and professionals to make an informed decision on treatment. In the current study, we investigated the risk of CVD in BC patients treated with versus without a radiation boost. In addition, we evaluated whether this relationship was modified by age.

## Methods

### Data sources

For the present study, we used data from the multicenter Bragatston cohort study [14]. In the Bragatston cohort patients with non-metastatic primary BC were included [14]. All BC patients treated with radiotherapy between 2005 and 2016 at the University Medical Center Utrecht were selected. In Bragatston, radiotherapy planning CT-scans were collected, subsequently, patients were linked with the Netherlands Cancer Registry to examine clinical characteristics, such as laterality, TNM stage, grade, receptor status, type of surgery and types of cancer treatment [15]. Data regarding a radiation boost were extracted from patient records by the radiotherapy department of the University Medical Center Utrecht. Guidelines for the dose of a radiation boost changed over the years [16], the different recommendations regarding radiotherapy regimens are presented in the supplemental methods 1. Guideline recommendations also changed over the years from recommending a boost for all patients younger than 50 years to a recommendation for all patients younger than 40 years. Patients were excluded if they were diagnosed with metastasized primary BC or pre-existing CVD, or if the baseline CT scan (for radiotherapy planning) was performed ≥ 1 year after BC diagnosis. Thoracic and coronary artery calcium calculation (TAC, CAC) is described in the supplemental methods 2 and described in a previous study [17]. 

Mortality and hospital admission data for CVD and cancer (recurrence) were obtained through linkage with national registries of Statistics Netherlands using a combination of the national personal identification number, sex, date of birth, and postal code for pseudonymization [18]. Data on hospitalization were obtained by linkage with the national basic registration hospital care, managed by Dutch Hospital Data. Hospital admission data included inpatient hospital care, day care and observations (≥ 4 h). Mortality data were obtained from the National Death Register, which contains information on primary cause of death from all deceased persons registered in the Netherlands. Statistics Netherlands uses the International Statistical Classification of Diseases and Related Health Problems, Tenth Revision (ICD-10) since 2013 for disease and cause of death classification. The classification of diseases and cause of death before 2013 were converted from ICD-9 to ICD-10. A waiver was provided for the Bragatston study by the Medical Research Ethics Committee of the University Medical Center Utrecht.

### Endpoint and follow-up

We followed patients for fatal and/or nonfatal CVD (I00-I99), IHD (I20-I25), conduction disorders (I44-I49), heart failure and cardiomyopathy (I50, I42, I43), and valvular disorders (I34-I37) [19]. The overarching CVD endpoint includes all previously listed types of CVD. Lymphedema (I88-I89) and varicose veins (I83-I86) were not considered as CVD outcomes. Competing events were related to malignancies defined by ICD-10 codes C00-C99, including recurrences and metastasis, and excluding nonmelanoma skin cancer (C44). Patients were followed from the acquisition of radiotherapy planning CT scan to the first occurrence of an endpoint, a censoring event or until the end of the follow-up period (31st of December 2018), whichever came first. A minimum follow-up period of two years was defined, as a latency period between radiotherapy and date of radiotherapy plus two years. CVD events during or shortly after radiotherapy (2 year latency period) are not likely to be related to the radiation.

### Statistics

Baseline characteristics were summarized as mean (standard deviation [SD]) or median (interquartile range [IQR]) in case of skewed distributions for continuous variables, and proportions for categorical variables. Incomplete recording of covariates for primary analysis were imputed using the method of multivariate imputation by chained equations (MICE) and generating five imputed datasets with 10 iterations each. Cardiovascular and specifically IHD event rates per 1000 person-years (PY) were calculated for patients with and without a radiation boost. Fine and Gray competing risk survival analyses were performed to evaluate the relationship between boost and CVD/IHD incidence rates in the presence of malignancies and other CVD as competing risks. Competing risk events included hospitalizations and mortality. Effect estimates are presented as a proportional subdistribution hazard ratio (sHR) with a 95% confidence interval (CI). Incidence was visualized with the Aalen-Johansen estimator of cause-specific cumulative incidence.

To address confounding, the influence of age at inclusion, coronary artery calcium, laterality and systemic treatments on the sHRs was evaluated. No data on traditional cardiovascular risk factors was available for analysis. Furthermore, an interaction between boost and age at inclusion was tested. In case of interaction with age an age-specific sHR was calculated within the observed age range in both boost and no boost groups (e.g. 25 to 75 years). The variable age was centered to each specific age that was analyzed, so age centered to 25 years to provide an exact age-specific sHR including 95% CI for patients of 25 years old. The analysis focused on specific age groups, ranging from 25 to 75 years, with intervals of 5 years.

Subgroup analysis were performed with event rates per 1000 PY and Fine and Gray competing risk survival analyses for laterality and chemotherapy to evaluate if the effect of boost differs within certain groups. All analyses were performed using R Statistical Software (version 3.6.2., R Foundation).

## Results

A total of 5260 BC patients were included, with a mean follow-up of 80.3 months (SD 37.1). Of all patients, 1917 (36.4%) received a radiation boost. Mean age at radiotherapy planning CT scan was 57.8 years (SD 10.7). Mean age of patients with and without a boost was 54.0 and 60.0, respectively (Table 1). Patients without a boost received both anthracycline chemotherapy and tamoxifen more often compared to patients with a boost, 31.8% vs. 10.0% for anthracycline and 36.8% vs. 8.9% for tamoxifen respectively. A total of 51.5% of patients had left-sided BC, the BC characteristics for tumor stage, lymph node status, differentiation grade and receptor status were comparable between patients treated with or without a boost.

**Table 1:** Baseline characteristics of the 5260 breast cancer patients receiving radiotherapy between 2005 and 2016, stratified for boost and without boost

Characteristic	Total	Radiotherapy	
		With boost	Without boost
N (%)	5260	1917 (36.4)	3343 (63.6)
Follow-up time, mean (SD), months	80.3 (37.1)	111.6 (32.8)	62.4 (25.8)
Age at diagnosis, mean (SD)	57.8 (10.7)	54.0 (8.8)	60.0 (11.0)
BMI, mean (SD)	27.1 (2.7)	27.1 (2.7)	27.5 (2.6)
Menopausal status, N (%)			
Premenopausal	1231 (23.4)	141 (7.4)	1090 (32.6)
Perimenopausal	262 (5.0)	138 (7.2)	124 (3.7)
Postmenopausal	3767 (71.6)	1638 (85.4)	2129 (63.7)
Laterality, N (%)			
Left-sided	2710 (51.5)	977 (51.0)	1733 (51.8)
Tumor stage, N (%)			
DCIS/T0	733 (13.9)	251 (13.1)	482 (14.4)
T1	3232 (61.4)	1237 (64.5)	1995 (59.7)
T2	1077 (20.5)	410 (21.4)	667 (20.0)
T3+	125 (2.4)	< 10 ^a^	< 10 ^a^
Unknown	93 (1.8)	< 10 ^a^	< 10 ^a^
Lymph nodes, N (%)			
Negative	3733 (71.0)	1402 (73.1)	2331 (69.7)
Positive	1415 (26.9)	501 (26.1)	914 (27.3)
Unknown	112 (2.1)	14 (0.7)	98 (2.9)
Differentiation grade, N (%)			
Well differentiated	1468 (27.9)	580 (30.3)	888 (26.6)
Moderately differentiated	1888 (35.9)	649 (33.9)	1239 (37.1)
Poorly differentiated	1388 (26.4)	536 (28.0)	852 (25.5)
Unknown / not reported	516 (9.8)	152 (7.9)	364 (10.9)
Receptor status, N (%) ^b^			
ER positive	4180 (79.5)	1645 (85.8)	2535 (75.8)
PR positive	3816 (72.5)	1642 (85.7)	2174 (65.0)
HER2 positive	1112 (21.1)	391 (20.4)	721 (21.6)
Triple negative	716 (13.6)	265 (13.8)	451 (13.5)
Surgery, N (%)			
Lumpectomy	4787 (91.0)	1899 (99.1)	2888 (86.4)
Mastectomy	459 (8.7)	16 (0.8)	443 (13.3)
Unknown	14 (0.3)	2 (0.1)	12 (0.3)
Chemotherapy, N (%)			
With anthracyclines	1253 (23.8)	191 (10.0)	1062 (31.8)
Without anthracyclines	758 (14.4)	589 (30.7)	169 (5.1)
None / Unknown	3249 (61.8)	1137 (59.3)	2112 (63.2)
Targeted therapy, N (%)			
Trastuzumab	367 (7.0)	121 (6.3)	246 (7.4)
None / Unknown	4893 (93.0)	1796 (93.7)	3097 (92.6)
Endocrine therapy, N (%)			
Aromatase inhibitors	113 (2.1)	32 (1.7)	81 (2.4)
Tamoxifen ^c^	1402 (26.7)	171 (8.9)	1231 (36.8)
Type of hormone therapy unknown	727 (13.8)	520 (27.1)	207 (6.2)
None / Unknown	3018 (57.4)	1194 (62.3)	1824 (54.6)
CAC score in Agatston units, N (%)			
0	3795 (72.1)	1404 (73.2)	2391 (71.5)
1–10	583 (11.1)	241 (12.6)	342 (10.2)
11–100	593 (11.3)	204 (10.6)	389 (11.6)
101–400	208 (4.0)	49 (2.6)	159 (4.8)
> 400	81 (1.5)	19 (1.0)	62 (1.9)
TAC in Agatston units, N (%)			
0	1845 (35.1)	717 (37.4)	1128 (33.7)
≥ 1	3415 (64.9)	1200 (62.6)	2215 (66.3)

Hormone receptor: ER: estrogen, PR: progesterone. If unknown was not reported, there were no missing data

HER2: human epidermal growth factor receptor 2, CAC: coronary artery calcium, TAC: thoracic aorta calcium

^a^ Subgroup counts below 10 are not reported in agreement with privacy regulations of Statistics Netherlands,

^b^ ER and PR were categorized as positive if ≥ 10% of tumor cells were positive, HER2 was categorized as positive for a 3+ score for immunohistochemistry test,

^c^ After tamoxifen treatment, 684 patients were treated with aromatase inhibitors or other types of hormone therapy

### Cardiovascular disease

**Fig. 1:**
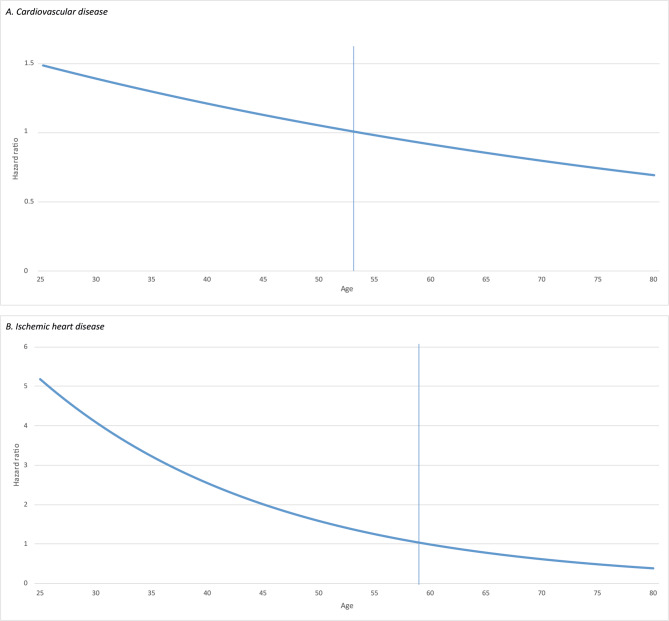
Visualization of the change in boost-HR for cardiovascular and ischemic heart disease, stratified for age. The line represents the change in HR with increasing age. The vertical line represents the mean age. (**A**) Cardiovascular disease. (**B**) Ischemic heart disease

A first event of either a CVD hospitalization or CVD-related mortality occurred in 365 (6.9%) patients (Table 2). Of the 30 patients who died of CVD, 13 had a prior hospitalization. The overall CVD incidence rate per 1000 PY was higher in patients without a boost compared to patients with a boost (11.5 vs. 8.2 per 1000 PY, respectively). The age-specific incidence rate per 1000 PY for patients with a boost compared to patients without a boost was higher in the age groups ‘< 50 years’ and ‘50 to 60 years’ (Tables 3 and Supplemental Fig. 1). Competing risk analysis showed a strong interaction between boost radiation and age, therefore age-specific sHR are presented. A relationship between boost and CVD was observed in patients younger than 50 years, although not statistically significant (Table 4). Patients of 25 years old had a sHR of 1.5 (95% CI 0.6–3.6) and this decreased to a sHR of 0.7 (95% CI 0.5–1.1) in 75-year old patients. The sHRs of the relationship between boost and CVD are stratified for all ages and presented in Fig. 1.

**Table 2:** Hospitalizations and mortality related to cardiovascular diseases and malignancies

	Boost	No boost		Total
N	1917	3343		5260
**Cardiovascular events, N (%)**				
All cardiovascular diseases	153 (8.0)	212 (6.3)		365 (6.9)
Ischemic heart disease	40 (2.1)	62 (1.9)		102 (1.9)
Heart failure	22 (1.1)	24 (0.7)		46 (0.9)
Conduction disorders	38 (2.0)	52 (1.6)		90 (1.7)
Valvular diseases	< 10 ^a^	< 10 ^a^		22 (0.4)
Cerebrovascular events	21 (1.1)	52 (1.6)		73 (1.4)
Vascular diseases	< 10 ^a^	< 10 ^a^		26 (0.5)
Other cardiovascular diseases	32 (1.7)	34 (1.0)		66 (1.3)
**Oncological events, N (%)**				
All malignancies	389 (20.3)	301 (9.0)		690 (13.1)
Breast cancer	231 (12.1)	159 (4.8)		390 (7.4)

Events include hospitalizations and mortality, ^a^ Subgroup counts below 10 are not reported in agreement with privacy regulations of Statistics Netherlands

**Table 3:** Incidence rates of all cardiovascular disease events and ischemic heart disease events, for patients with and without boost stratified for age

	N total	Events N (%)	Person-years	Incidence rate per 1000 person-years	Incidence rates per 1000 person-years, stratified for age		
					< 50 years N^nb^ = 649 N^b^ = 608	50–60 years N^nb^ = 919 N^b^ = 863	> 60 years N^nb^ = 1775 N^b^ = 446
**Cardiovascular disease**							
No boost	3343	201 (6.0)	17,545	11.5	4.0	4.7	17.0
Boost	1917	147 (7.7)	17,913	8.2	5.0	8.0	13.7
**Ischemic heart disease**							
No boost	3343	51 (1.5)	17,545	2.9	0.3	0.4	4.9
Boost	1917	35 (1.8)	17,913	2.0	1.0	2.4	2.4

N^nb^: Number of patients in no boost group, N^b^: Number of patients in boost group

**Table 4:** Fine and Gray competing risk survival analyses of boost radiotherapy and cardiovascular disease or ischemic heart disease events

	N	Competing risk events N (%)	Competing risk models, sHR (95% CI) *							
			Crude	Age 25	Age 35	Age 39	Age 45	Age 55	Age 65	Age 75
**Cardiovascular disease** ^a^										
No boost	3343	268 (8.0)	1 [Ref]	1 [Ref]	1 [Ref]	1 [Ref]	1 [Ref]	1 [Ref]	1 [Ref]	1 [Ref]
Boost	1917	345 (18.0)	0.4 (0.3–0.6)	1.5 (0.6–3.6)	1.3 (0.7–2.5)	1.2 (0.7–2.1)	1.1 (0.7–1.8)	1.0 (0.7–1.3)	0.9 (0.7–1.1)	0.7 (0.5–1.1)
**Ischemic heart disease** ^b^										
No boost	3343	418 (12.5)	1 [Ref]	1 [Ref]	1 [Ref]	1 [Ref]	1 [Ref]	1 [Ref]	1 [Ref]	1 [Ref]
Boost	1917	457 (23.8)	0.4 (0.2–0.7)	5.1 (1.2–22.6)	3.2 (1.04–9.8)	2.8 (1.002–7.7)	2.0 (0.9–4.4)	1.2 (0.7–2.1)	0.8 (0.5–1.3)	0.5 (0.2–1.02)

* The age-specific models are corrected for competing risks, age-centered and the interaction with age-centered. Hazard ratios are calculated by centering for each age, therefore the hazard ratios are specifically for the presented ages and not for an age group. Analysis was corrected for the following confounding factors: coronary artery calcium, laterality, chemotherapy and endocrine therapy. ^a^ Competing risk: malignancies, ^b^ Competing risk: malignancies and other cardiovascular diseases. Proportion competing risk events is calculated with subgroup totals, for no boost *N* = 3343 and for boost *N* = 1917.

### Ischemic heart disease

The overall incidence rate per 1000 PY of IHD was slightly higher in patients without a boost compared to patients with a boost (event rate 2.9 vs. 2.0 per 1000 PY). The age-specific incidence rates per 1000 PY in boost patients compared to patients without a boost were higher in the age groups ‘<50 years’ (1.0 vs. 0.3 per 1000 PY) and ’50 to 60 years’ (2.4 vs. 0.4 per 1000 PY; Table 3). Competing risk analysis showed a strong interaction between boost radiation and age, therefore age-specific sHR are presented. A radiation boost was significantly associated with IHD incidence in patients up to the age of 39 years in the survival models. The sHR ranged from 5.1 (95% CI 1.2–22.6) in 25-year old patients to a sHR of 0.5 (95% CI 0.2–1.02) in 75-year old patients (Tables 4 and Fig. 1). The effect of a radiation boost seems to be amplified in patients who received chemotherapy with a sHR of 2.9 (95% CI 0.8–10.5), compared to patients without a radiation boost although not statistically significant (Supplemental Table 2). No significant association with IHD incidence was observed in boost patients receiving left-sided radiotherapy (sHR 1.1, 95% CI 0.6–2.3), compared to patients without a boost.

## Discussion

This is the first study evaluating the effect of a radiation boost on cardiovascular and specifically IHD incidence in BC patients. An interaction between boost and age was observed, therefore cardiovascular and IHD risks were determined for specific age groups. This study indicates that, although numbers were low and CIs were wide, a significant increase in IHD risk in boost patients younger than 40 years could be observed. No significant IHD risk for patients older than 40 years was observed, we hypothesize that this might be related to the presence of other cardiac risk factors which we could not correct for in this study. Absolute risk of IHD remained low in all age groups.

Radiotherapy is an important treatment option in BC patients to reduce the risk of local recurrences. Since it became clear that thoracic radiotherapy is a risk factor for CVD, new techniques were developed to reduce radiation dose on the heart, for example with intensity modulated radiotherapy (IMRT) or volumetric modulated arc therapy (VMAT) in combination with breath-hold techniques [20]. In the Netherlands, radiotherapy is now given with protons in patients with a MHD above an established threshold in the photon plan depending on risk factors for CVD and age [20, 21]. In line with the results of this study specifically IHD risk increases, a previous study showed a 7.4% linear increase in IHD risk per one Gy increase of the MHD [4, 5, 22]. It should be noted that without cardiac sparing techniques, the patients in these studies received an average MHD of 20 Gy [22]. With cardiac sparing techniques, patients receive a significantly lower dose on the heart. A study in BC patients evaluating the radiation dose to the heart showed that deep inspiration breath-hold can even reduce MHD up to 48% [6]. Although these techniques significantly reduce MHD, the anterior part of the heart could still receive a relatively high dose. A previous study showed a mean dose on the LAD of 15.68 Gy in left-sided BC patients while MHD was only 2.95 Gy [7]. Additionally, it seems that even low MHD of 0.5 Gy can accelerate the atherosclerotic process, as seen in animal models [23]. A prospective patient study by Demissei et al. showed an increase in cardiovascular inflammatory markers during radiotherapy with an average MHD of 2.1 Gy [24]. This suggests that even low radiation doses could have a negative impact on cardiovascular health, and specifically increase IHD risk. Considering the contemporary radiation regimens with cardiac sparing techniques, evaluating the radiation dose on cardiac substructures is recommended as it might be a more accurate predictor for IHD risk, compared to MHD [25]. 

A radiation boost improves local control of BC after breast conserving therapy. Especially younger patients (≤ 40 years) seem to benefit from a reduced recurrence rate after a radiation boost [11]. Even though a boost reduces the absolute risk of local recurrences, it does not improve the 10-year survival rate for these patients [11]. A boost, however, could affect the risk of IHD, as seen in this study. Therefore, the benefit of a radiation boost on local recurrence is a topic of debate, specifically for young women the introduction of routine hormone therapy might have reduced the added beneficial influence of receiving a boost [26]. The results of this study highlight the need of evaluating the beneficial effects of a boost, as well as toxicity. Overall, young female patients have a low pre-treatment probability for coronary artery disease (< 1% 10-year risk) [27], but more young patients who received a boost developed IHD compared to patients who did not receive a radiation boost. Moreover, the CVD risk seems to be more pronounced in patients receiving systemic cancer therapy, such as cyclophosphamide and anthracyclines [28, 29]. Therefore, for patients with a high baseline IHD risk – based on patient- and treatment-specific risk factors – a possible negative impact of a radiation boost on IHD incidence might outweigh the benefit on local control of BC. This discussion, however, should be patient-tailored using cardiovascular risk stratification before cancer treatment. Current recommendations for risk stratification and monitoring include controlling cardiovascular risk factors and screening with multimodality imaging every five years in patients with a high CVD risk [30, 31]. In current literature, there are recommendations for the treatment of IHD after thoracic irradiation. In these patients, a percutaneous coronary intervention is preferred over coronary artery bypass grafting since the latter is often complicated by inadequate bypass targets, a high rate of compound procedures and poor wound healing [5]. 

### Study limitations

In this study, no large differences were observed in tumor characteristics in BC patients. This can be explained by the age differences between the groups: younger age is the most important risk factor for local recurrence, therefore younger patients treated with breast conserving therapy are more likely to receive a boost compared to older patients with comparable BC characteristics (Supplemental Table 3). Boost patients often have more aggressive tumors and in this study the patients have more malignancy-related events during follow-up and also a higher IHD risk, future studies are needed to evaluate a potential link between cancer biology and cardiovascular diseases [32]. This study had a follow-up period of on average 6.7 years. Cardiotoxic effects of radiotherapy can be detected 2–15 years after treatment, suggesting that a longer follow-up period would result in a larger number of IHD events. A longer and equal between-groups follow-up period could have resulted in finding a different risk. A limitation of our study was the unavailability of data on cardiovascular risk factors, and therefore, survival models could not be corrected for risk factor status. Although, patients with an increased IHD risk were younger than 40 years, Dutch risk factor data shows that 100% of the population aged younger than 44 have a 10-year CVD risk of less than 5% [33]. Therefore, we expect that the number of traditional cardiovascular risk factors in this population would be low. The non-significant effect of boost on IHD risk in patients older than 40 years might be related to an already increased baseline cardiovascular risk in these patients, potentially the additional risk of a radiation boost could not be observed without a correction for cardiovascular risk factors.

In addition, patients with cardiovascular diseases and risk factors are less likely to receive a boost at an older age. Although patients without a boost received anthracycline and tamoxifen therapy more often than boost patients, anthracycline therapy is a known risk factor for CVD and tamoxifen has a potential cardioprotective effect. In this study we corrected for these potential confounding factors, but correction for anthracycline or tamoxifen did not significantly change the results in this study. Although in subgroup analysis, patients receiving anthracycline chemotherapy and a radiation boost appeared to have a higher risk but this remained non-significant, this could potentially be related to a lack of power in the subgroup analysis. Therefore, age-specific hazards were not presented for these age groups.

## Conclusions

This study indicates an association between radiation boost and the development of IHD in patients younger than 40 years, although due to the low numbers in younger age groups CIs were wide. Additional treatment of chemotherapy might increase the IHD risk. A patient-tailored risk-benefit ratio might be important for young BC patients for shared decision making in clinical practice, to protect both cancer-related and cardiovascular prognosis. Evidence regarding the benefits and the adverse cardiovascular effects of a radiation boost could guide shared decision making for radiotherapy treatment.

### Clinical perspectives

*Competency in medical knowledge*: In breast cancer patients receiving a radiation boost a significant relationship was observed with the risk for ischemic heart disease in patients younger than 40 years.

*Translational outlook*: Future prospective studies should evaluate cardiovascular disease risk in breast cancer patients treated with contemporary radiation regimen and take traditional cardiovascular risk factors and radiation dose on cardiac structures into account.

### Electronic supplementary material

Below is the link to the electronic supplementary material.

Supplementary Material 1

## Data Availability

The data that support the findings of this study are available from Statistics Netherlands but restrictions apply to the availability of these data, which were used under license for the current study, and so are not publicly available.

## Abbreviations

BC: Breast cancer
CVD: Cardiovascular disease
ICD-10: International Statistical Classification of Diseases and Related Health Problems, Tenth Revision
IHD: Ischemic heart disease
IMRT: Intensity modulated radiotherapy
IQR: Interquartile range
MHD: Mean heart dose
PY: Person years
sHR: Subdistribution hazard ratio
VMAT: Volumetric modulated arc therapy
