# Supplementary material for: Competing risk analysis of cardiovascular disease risk in breast cancer patients receiving a radiation boost
Source: Cardiooncology. 2024 Feb 9;10:7. doi: 10.1186/s40959-024-00206-4 (PMC10854185; doi:10.1186/s40959-024-00206-4)

**Supplemental files**

**Manuscript:** Competing risk analysis of cardiovascular disease risk in breast cancer patients receiving a radiation boost

**Table of content**

Supplemental methods 1. Boost dose recommendations between 2005 and 2016

Supplemental methods 2. Coronary artery calcium score calculation

Supplemental table 1. Cardiovascular disease and malignancy ICD-10 codes

Supplemental table 2. Fine and Gray competing risk survival analyses of boost radiotherapy

and cardiovascular disease or ischemic heart disease, stratified for chemotherapy and laterality

Supplemental table 3. Proportions for tumor stage and age distribution between boost and

without boost groups

Supplemental figure 1. Aalen-Johansen plot CVD incidence in patients with and without

boost, corrected for malignancies as competing risk and uncorrected

for baseline and treatment covariates.

**Supplemental methods 1: Boost dose recommendations between 2005 and 2016**

In the Netherlands, the national recommendations for radiotherapy regimen differed over the years. Before 2011, it was 25 fractions of 2 Gy whole breast irradiation with a sequential boost of 8 fractions 2 Gy (R0, radical resection with microscopic tumor clearance) or 10 fractions of 2 Gy (R1, focal resection with microscopic tumor infiltration). Between 2011 and 2016, it was 21 fractions of 2.17 Gy whole breast irradiation with 21 fractions of 2.66 Gy simultaneous integrated boost (SIB) for R0 and 23 fractions of 2.03 Gy whole breast irradiation with 23 fractions of 2.66 Gy SIB for R1. If SIB was not possible, it was 16 fractions of 2.66 Gy whole breast irradiation with 5 fractions (R0) or 7 fractions (R1) of 2.66 Gy sequential boost.

**Supplemental methods 2: Coronary artery calcium calculation**

For coronary artery calcium (CAC) calculation, an artificial intelligence based algorithm automatically segmented each cardiac structure, for which a standard radiotherapy planning thoracic CT scan was used (no contrast-enhancement, no ECG-triggering, free breathing, 120 kVp, 0.78-1.37 mm in-plane resolution, 3 mm slice thickness and 3 mm slice spacing). The cardiac structures included atria and ventricles, aorta, pulmonary artery and coronary arteries (Left anterior descending [LAD] coronary artery incl. left main [LM], Right circumflex [RCx], Right coronary artery [RCA]). The presence and amount of CAC was quantified using a deep-learning calcium scoring algorithm and represented as Agatston score. Agatston score was calculated by multiplying the calcification area (in mm^2^) by the weight (1, 130-199 Hounsfield Units [HU]; 2, 200-299 HU; 3, 300-399 HU; 4, >399 HU), which was determined by the maximum density of the area and summing the lesion scores over slices. A minimal lesion definition of 1.5mm^3^ was maintained to eliminate noise. Patients were categorized into 5 categories for CAC score: 0, 0 to 10, 11 to 100, 101 to 400, >400 Agatston units.

| **Supplemental table 1. Cardiovascular disease and malignancy ICD-10 codes** | |
| --- | --- |
|  | **ICD-10 codes** |
| **Cardiovascular events** |  |
| All cardiovascular diseases | I00-199, except I88-I89 (lymphedema) and I83-86 (varicose veins) |
| Ischemic heart disease | I20-25 (e.g. myocardial infarction and angina pectoris) |
| Heart failure | I42, I43 and I50 (heart failure and cardiomyopathies) |
| Conduction disorders | I44-I49 (e.g. atrial fibrillation) |
| Valvular disease | I34-I37 (nonrheumatic valve disorders) |
| Cerebrovascular diseases | I60-I69 (e.g. intracranial hemorrhage and cerebral infarction) |
| Diseases of the arteries,  arterioles and capillaries | I70-I79 (e.g. aortic aneurysm and arterial embolism or thrombosis) |
| Other cardiovascular diseases | I10-I15, I30-I41 and I51 (e.g. hypertensive diseases and infectious diseases) |
| **Oncological events** |  |
| All malignancies | C00-C97 |
| Breast cancer | C50 |

| Supplemental table 2. Fine and Gray competing risk survival analyses of boost radiotherapy and cardiovascular disease or ischemic heart disease, stratified for chemotherapy and laterality | | | | | | |
| --- | --- | --- | --- | --- | --- | --- |
|  | **N** | **Events**  **N (%)** | **Person-years** | **Incidence rate per 1000 person-years** | **Competing risk events**  **N (%)** | **Competing risk model ^d^**  **sHR (95% CI)** |
| Cardiovascular disease |  |  |  |  |  |  |
| Chemotherapy |  |  |  |  |  |  |
| No boost | 1231 | 38 (3.1) | 6165 | 6.2 | 95 (7.7) | 1 [Ref] |
| Boost | 780 | 50 (6.4) | 7313 | 6.8 | 141 (18.1) | 1.0 (0.6 – 1.6) |
| No chemotherapy |  |  |  |  |  |  |
| No boost | 2112 | 163 (7.7) | 11380 | 14.3 | 173 (8.2) | 1 [Ref] |
| Boost | 1137 | 97 (8.5) | 10600 | 9.2 | 204 (17.9) | 0.9 (0.6 – 1.3) |
| Left-sided radiation |  |  |  |  |  |  |
| No boost | 1733 | 104 (6.0) | 9128 | 11.4 | 149 (8.6) | 1 [Ref] |
| Boost | 978 | 81 (8.3) | 9213 | 8.8 | 167 (17.1) | 1.0 (0.7 – 1.5) |
| Right-sided radiation |  |  |  |  |  |  |
| No boost | 1610 | 97 (6.0) | 8417 | 11.5 | 119 (7.4) | 1 [Ref] |
| Boost | 939 | 66 (7.0) | 8700 | 7.6 | 178 (19.0) | 0.9 (0.6 – 1.3) |
| Ischemic heart disease |  |  |  |  |  |  |
| Chemotherapy |  |  |  |  |  |  |
| No boost | 1231 | <10 ^c^ | 6165 | 0.6 | 129 (10.5) | 1 [Ref] |
| Boost | 780 | 12 (1.5) | 7313 | 1.6 | 179 (22.9) | 2.9 (0.8 – 10.5) |
| No chemotherapy |  |  |  |  |  |  |
| No boost | 2112 | 47 (2.2) | 11380 | 4.1 | 289 (13.7) | 1 [Ref] |
| Boost | 1137 | 23 (2.0) | 10600 | 2.2 | 278 (24.5) | 0.8 (0.4 – 1.4) |
| Left-sided radiation |  |  |  |  |  |  |
| No boost | 1733 | 27 (1.6) | 9128 | 3.0 | 226 (13.0) | 1 [Ref] |
| Boost | 978 | 21 (2.1) | 9213 | 2.3 | 227 (23.2) | 1.1 (0.6 – 2.3) |
| Right-sided radiation |  |  |  |  |  |  |
| No boost | 1610 | 24 (1.5) | 8417 | 2.9 | 192 (11.9) | 1 [Ref] |
| Boost | 939 | 14 (1.5) | 8700 | 1.6 | 230 (24.5) | 1.0 (0.5 – 2.1) |
| Models are corrected for competing risks, age-centered around the mean and the interaction with age centered. ^a^ Competing risk: malignancies, ^b^ Competing risk: malignancies and other cardiovascular diseases. ^c^ Subgroup counts below 10 are not reported in agreement with privacy regulations of Statistics Netherlands. ^d^ The interaction component for boost with age centered around the mean was not significant, which could be related to the reduction in sample size. Presented hazard ratios are for the mean age of 58 years. | | | | | | |

| **Supplemental table 3. Proportions for tumor stage and age distribution between boost and without boost groups** | | | | | | | | | | | | | | |
| --- | --- | --- | --- | --- | --- | --- | --- | --- | --- | --- | --- | --- | --- | --- |
|  | **DCIS/T0** | |  | **T1** | |  | **T2** | |  | **N0** | |  | **N+** | |
|  | **Boost** | **No boost** |  | **Boost** | **No boost** |  | **Boost** | **No boost** |  | **Boost** | **No Boost** |  | **Boost** | **No Boost** |
| **Age groups, %** |  |  |  |  |  |  |  |  |  |  |  |  |  |  |
| Age <40 | * | 0.6 |  | 3.5 | 1.4 |  | 1.1 | 1.0 |  | 3.4 | 2.0 |  | 1.5 | 0.7 |
| Age 40-49 | 2.4 | 1.8 |  | 15.6 | 8.5 |  | 8.3 | 4.2 |  | 17.4 | 8.3 |  | 8.7 | 6.8 |
| Age 50-59 | 7.2 | 4.6 |  | 29.8 | 15.7 |  | 7.7 | 5.5 |  | 34.3 | 18.9 |  | 10.1 | 7.3 |
| Age 60-69 | 2.8 | 4.8 |  | 13.6 | 21.5 |  | 3.0 | 5.1 |  | 15.0 | 25.3 |  | 4.6 | 6.0 |
| Age ≥ 70 | * | 2.5 |  | 2.0 | 12.5 |  | 1.1 | 4.2 |  | 3.0 | 15.3 |  | * | 3.6 |
| * Subgroup proportions were based on absolute numbers below 10, these values are not reported in agreement with privacy regulations of Statistics Netherlands.  Percentages are calculated using total group counts for boost (N = 1917) and for no boost (N = 3343), due to missingness totals don’t add up to 100%. | | | | | | | | | | | | | | |

**Supplemental figure 1. Aalen-Johansen plot CVD incidence in patients with and without boost, corrected for malignancies as competing risk and uncorrected for baseline and treatment covariates**


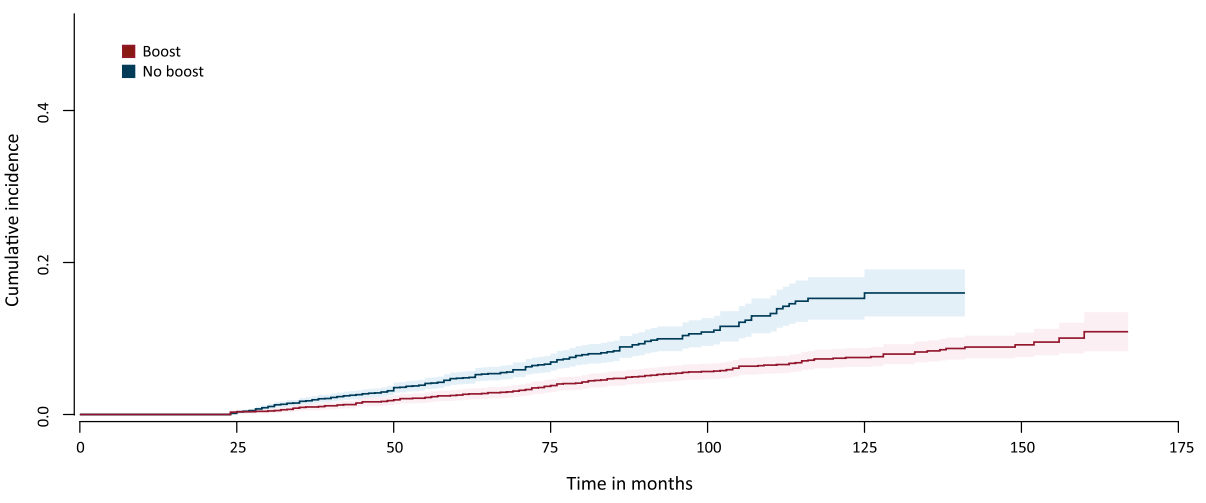

Supplement: Supplementary file 1 — Supplementary Material 1 [file 40959_2024_206_MOESM1_ESM.docx]
